# Interaction between hypertension and frailty and their impact on death risk in older adults: a follow-up study

**DOI:** 10.1186/s12877-024-04793-w

**Published:** 2024-02-24

**Authors:** Jing Shi, Yongkang Tao, Shuqiang Chen, Ziyi Zhou, Li Meng, Chunbo Duan, Baiyu Zhou, Pulin Yu

**Affiliations:** 1 The Key Laboratory of Geriatrics, Beijing Institute of Geriatrics, Institute of Geriatric Medicine, Chinese Academy of Medical Sciences, Beijing Hospital/National Center of Gerontology of National Health Commission, Beijing, 100730 China; 2 Department of Gastroenterology, China-Japan Friendship Hospital, Beijing, 100029 China

**Keywords:** Frailty, Hypertension, Mortality, Older adults, Follow-up study

## Abstract

**Background:**

Hypertension and frailty often occur concurrently, exhibiting increasing prevalence in the older population. In this study, we analyzed the frailty status among older adults with hypertension and the impact of their interaction on death risk.

**Method:**

This prospective cohort survey study included data from older people in an urban community in Beijing collected between 2009 and 2020 using the cluster random sampling method. The participants were older adults who were ≥ 60 years old at the time of investigation and had lived at the place of investigation for > 1 year. The survey variables comprised those related to health and frailty status assessed during the 2009 baseline survey, along with death-related information as outcome variables in 2020. Additionally, a frailty index (FI) model was used to examine the frailty status among the older adults at baseline. The effects of hypertension prevalence on the age-related frailty changes as well as on mortality for varying degrees of frailty were further analyzed. Lastly, Cox regression and Kaplan–Meier curves were applied to evaluate the impact of the interaction between hypertension and frailty on death risk.

**Results:**

Ultimately, 1197 older individuals aged between 60 and 101 years(average age at baseline: 74.8 ± 8.6 years) were included .Among them, 475 individuals were men (mean age:74.8 ± 8.8 years), and 722 were women (mean age:74.8 ± 8.4 years).Frailty was identified in 151 individuals, leading to a prevalence rate of 12.6%(151/1197),while hypertension was detected in 593 (prevalence rate:49.5% [593/1197]).A total of 443 deaths were recorded by 2020, resulting in a mortality rate of 37.0% (443/1197).Moreover, FI values and mortality rates were higher at any age in older adults with hypertension compared with those without hypertension. Survival time analysis showed that the median survival time of older adults with hypertension and frailty was the shortest (39.0[95%*CI*: 35.6–42.3] months)when compared with that of older adults without hypertension but with frailty (52.9 [95%*CI*: 46.6-59.3] months), those with hypertension but without frailty (102.7 [95%*CI*: 98.7–106.8] months), and those without hypertension and frailty (127.9 [95%*CI*: 113.5–134.7] months),with log-rank *x*^*2*^ = 999.686 and *P* < 0.001. Furthermore, Cox regression results demonstrated that older adults with hypertension and frailty had the highest death risk when compared with that of older adults without hypertension and frailty (*HR* = 1.792, *P* < 0.001), those without hypertension but with frailty (*HR* = 1.484, *P* < 0.001), and those with hypertension but without frailty (*HR* = 1.406, *P* = 0.005).

**Conclusion:**

Frailty is prevalent among older adults with hypertension; however, older adults with both hypertension and frailty have a relatively higher mortality risk. Therefore, screening and assessment of frailty in the older population with hypertension are crucial for its early identification, thereby enabling timely and appropriate interventions to prevent or delay the adverse effects of this concurrent condition.

**Supplementary Information:**

The online version contains supplementary material available at 10.1186/s12877-024-04793-w.

## Background

Population aging and increasing life expectancy have led to a rise in the incidence of chronic diseases and functional impairments, posing significant challenges to society and the healthcare system. Among the major health problems faced by older adults, hypertension shows a strong association with adverse health outcomes, including mortality [1]. Moreover, the hypertension prevalence rate increases with age, demonstrating an upward trend worldwide [2, 3]. In the context of aging, the risk of hypertension co-occurring with frailty advances with age, leading to a growing prevalence of this concurrent condition in older adults [4]. Frailty is a geriatric syndrome involving a decline in multiple physiological reserves of the body, along with decreased resistance to stressors, heightened vulnerability, and diminished ability to maintain homeostasis. Frailty is also linked to an increased risk of disability, hospitalization, and death in older adults. The prevalence rate of frailty in older adults is 2.3%–20.2%, indicating an escalating trend with age [5, 6].

The growing recognition of frailty as a health condition has resulted in a gradual increase in research investigating the relationship between frailty and hypertension. Frailty and hypertension in older adults have been suggested to have a reciprocal interaction. For example, chronic diseases such as hypertension are considered significant risk factors for frailty [7], with hypertension-related cardiovascular complications being reported as contributing factors to frailty occurrence [8]. Conversely, frailty is a suggested risk factor for cardiovascular diseases, including hypertension [9]. The effects of coexisting frailty and hypertension have also been demonstrated in terms of adverse outcomes, with a few studies indicating that patients with hypertension accompanied by frailty experience a significant increase in the risk of death [10, 11]. Therefore, strengthening the understanding of frailty in older adults with hypertension may be a crucial aspect of hypertension management. Correspondingly, researchers in developed countries have emphasized monitoring, assessing, and preventing frailty in patients with hypertension [12–15]. However, only a few studies have been conducted on the interaction between hypertension and frailty, as well as its impact on long-term mortality. Although the community health services in China, which has an expanding population of older adults with hypertension living in the community, have prioritized the health management of the older population with hypertension, frailty is still not routinely screened for in this population [16]. Therefore, this study aimed to analyze the prevalence of frailty and hypertension among older adults in an urban community in Beijing, as well as explore their interaction and its impact on 11-year mortality. We hope that our study will provide a solid basis for enhancing the management of frailty in older adults with hypertension, thereby reducing frailty-associated adverse health outcomes.

## Materials and methods

### Research participants

This study is a secondary analysis of the Health Status and Fall Status Follow-up Survey database, a representative cohort of urban community-dwelling individuals aged ≥ 60 years in Beijing. In this study, the participants were from the sample population in the 2009 baseline survey, with death events in this cohort representing the outcome variables collected during the follow-up survey in 2020.The 2009 baseline survey was conducted in a community under the jurisdiction of a street office in Dongcheng District, Beijing, which was used as the survey site. Furthermore, a cluster random sampling method was employed to randomly select four neighborhood committees from the eleven neighborhood committees under the jurisdiction of the street office. Additionally, cluster sampling was conducted to investigate the adults aged ≥ 60 years by considering the residential buildings as units. The inclusion criteria were as follows: individuals who were ≥ 60 years old at the time of investigation (since individuals aged ≥ 60 years are regarded as older adults in China [17]) and had lived for > 1 year at the place of investigation. The exclusion criteria were as follows: older adults who did not live in the investigation area during the investigation period or those who could not cooperate in completing the investigation items. A total of 1578 older residents were eligible for the 2009 baseline survey. Among them, 37 individuals refused to be interviewed, while 63 were not available at their residences during the investigation period. Ultimately, 1478 older adults were surveyed, yielding a response rate of 93.7%. In 2020, a population follow-up of the 1478 older adults from the baseline survey was conducted to collect death information. By 2020, 232 older adults were lost to follow-up, with a loss rate of 15.7% (232/1478). All individuals lost to follow-up had left or relocated from the place of investigation, with a mean age of (68.2 ± 3.6) years and consisting of 108 males (46.6%) and 124 females (53.4%). Consequently, 1246 participants were selected for the analysis, comprising 519 males (41.7%) and 727 females (58.3%) with a mean age of (72.1 ± 4.5) years. The mean age of the older adults lost to follow-up was lower than those of the included older adults (*t* = 12.148, *P* < 0.001), but the gender difference was not statistically significant (*x*^*2*^ = 1.921, *P* = 0.166). All respondents provided a signed informed consent form.

### Questionnaire content and assessment of frailty

In this study, the sample population was administered a standard questionnaire that was finalized and validated after several rounds of expert discussions. The respondent data collected in the questionnaire were as follows: demographic characteristics (age, gender, education level, and marital status), health physical status (vision, hearing, and walking balance function), diseases (including hypertension) and medication, activities of daily living (ADL) and instrumental activities of daily living (IADL) [18], cognition and emotion (memory loss, emotional instability, and Mini-Mental State Examination [MMSE] assessment) [18], depression (Center for Epidemiologic Studies Depression [CES-D] scale) [18], and comprehensive geriatric assessment (falls, urinary incontinence, pain, constipation, weight loss, sleep disorder, and use of sleep aids).Moreover, diseases were considered only if diagnosed in hospitals, and respondents who self-reported their symptoms or measured their blood pressure at home without a precise diagnosis were excluded from the analysis. Frailty was assessed using the frailty index (FI) model developed by the research team of Canadian geriatrician Professor Kenneth Rockwood [19], which was scored using items included in the questionnaire. The FI model is based on the accumulation of health deficits, providing a quantitative description of the degree of individual frailty [19]. A total of 36 variables were selected as the health deficit indicators to calculate FI according to the previously mentioned criterion of health deficits, with the variables being assigned into categories based on the variable types [20]. As shown in Additional file 1: Table 1, the variables were grouped as follows: comprehensive geriatric assessment (seven items), vision and hearing assessment (two items), walking balance function (six items), diseases and medication (fifteen items), ADL (two items), cognition and emotion (three items), and depression (one item). In our study, the FI value was calculated only for the baseline sample. The formula for calculating FI was as follows: FI = number score of existing health deficits/total number of items considered health deficits (i.e., 36). The FI values ranged from 0 to 1, with larger values indicating more health deficits and a greater degree of frailty. Frailty was defined as FI ≥ 0.2, based on the grading method recommended by Searle et al. [20].

### Definition of follow-up outcomes

Mortality status in this cohort was recorded during the follow-up in 2020 as outcome variables, including death status (yes or no) and time of death. The information concerning deaths was obtained by staff via family members, local neighborhood committees, or local public security agencies. A standard method was used to calculate the precise follow-up time. In this method, the follow-up time for a study participant who died during the follow-up was calculated as “date of death − baseline date,” whereas the follow-up time when no death occurred was estimated as “last follow-up date − baseline date.”

### Statistical analysis

All data analyses and plotting were performed using SPSS 24.0 and Matlab 2020. The measurement data were expressed as mean ± standard deviation (x ± s), with variance analysis for comparisons among multiple groups. The enumeration data were presented as the number of participants (percentage), with the *x*^*2*^ test for group comparisons. A multiple imputations approach using the Markov Chain Monte Carlo method was used to fill in the missing values of the data set [21]. Further, nonlinear regression techniques were employed to fit age-specific FI values as a function of age (an exponential function) and to fit the probability of death as a function of the FI (a logistic function) between older people with and without hypertension. Additionally, the Kaplan–Meier method was applied to plot survival curves to analyze the impact of varied hypertension and frailty status on the survival time in older adults across different age groups, followed by a log-rank test. Lastly, the Cox regression model was utilized to assess the impact of combined hypertension and frailty on death risk in older adults across different age groups, with adjustment for statistically significant variables in the univariate analysis and exclusion of those used to calculate the FI values. Statistical significance was set at *P* < 0.05.

## Results

### Baseline data of older adults with different hypertension and frailty status

**Fig. 1:**
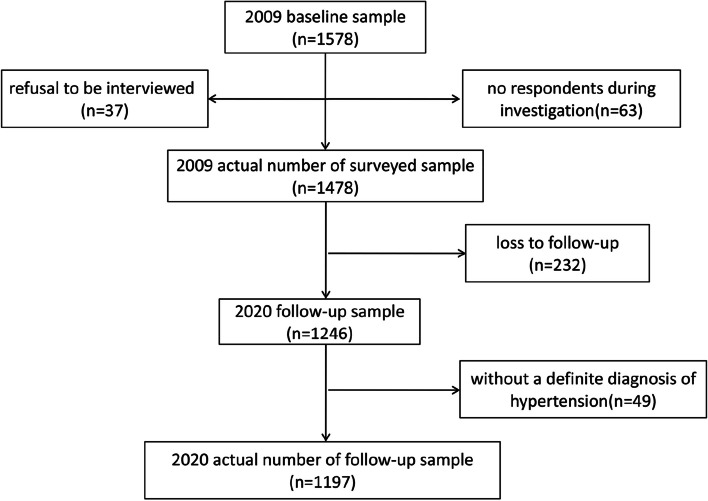
Flowchart of the enrolled sample and study design

Among the selected 1246 older adults, 49 were excluded because they had self-reported symptoms or measured their blood pressure at home without a precise diagnosis, eventually leading to 1197 individuals in the analysis (Fig. 1). The age of the included 1197 older adults ranged from 60 to 101 years, with a mean age of 74.8 ± 8.6 years and consisting of 475 men (mean age:74.8 ± 8.8 years) and 722 women (mean age:74.8 ± 8.4 years). By 2020, 443 deaths had occurred, resulting in a death rate of 37.0% (443/1197). Furthermore, these deaths were reported in 196 men (41.3%) and 247 women (34.2%), indicating that men had a higher death rate than women (*x*^*2*^ = 6.113, *P* = 0.013). In the case of hypertension incidence, 593 cases were observed, leading to a prevalence rate of 49.5% (593/1197).Moreover, hypertension was observed in 41.7% of men (198/475) and 54.7% of women (395/722), suggesting a higher prevalence of hypertension in women than in men (*x*^*2*^ = 19.444, *P* < 0.001). In terms of frailty, 151 cases were confirmed, showing a prevalence rate of 12.6%.Additionally, frailty was found in 9.1% of men (43/475) and 15% of women (108/722), implying that women had a higher prevalence of frailty than men (*x*^*2*^ = 9.065, *P* = 0.003). Further, the prevalence rate of frailty among older adults with hypertension was 15.0% (89/593), while that in older adults without hypertension was lower at 10.3% (62/604) (*x*^*2*^ = 6.108, *P* = 0.013). The comparison of the baseline data among older adults with different hypertension and frailty status showed that the higher the age of the individuals, the more likely they were to experience hypertension combined with frailty. Furthermore, the proportion of women with both hypertension and frailty was greater than that of men with this concurrent condition. Similarly, widowed older adults and those with a low education level exhibited a significantly higher proportion of hypertension accompanied by frailty. In the case of health-related indicators, older people with three or more chronic diseases and those with a higher number of medication types had a higher prevalence of hypertension with frailty. Moreover, older adults with different hypertension and frailty status demonstrated significant differences in their ADL, IADL, MMSE, and CES-D scores. In particular, the functional decline (ADL and IADL), cognitive decline (MMSE), and the worse symptoms of depression (CES-D) were closely related to hypertension and frailty status among older adults (all *P* < 0.05).Finally, older adults with both hypertension and frailty demonstrated a significantly higher death rate (see Table 1 for complete information).

**Table 1:** Comparison of the characteristics of the sample population grouped according to hypertension and frailty status

Variables	No hypertension and no frailty (*n* = 542)	Hypertension and no frailty (*n* = 504)	No hypertension but frailty (*n* = 62)	Hypertension and frailty (*n* = 89)	F/x^2^	* P*-value
Age(x ± s)	72.5 ± 8.6	75.0 ± 7.6	82.7 ± 7.5	82.2 ± 6.7	60.976	< 0.001
Gender,n(%)					26.883	< 0.001
Men	256(47.2)	176(34.9)	21(33.9)	22(24.7)		
Women	286(52.8)	328(65.1)	41(66.1)	67(75.3)		
Education level,n(%)					98.233	< 0.001
Primary school	144(26.6)	185(36.7)	43(69.4)	63(70.8)		
Junior high school	165(30.4)	137(27.2)	9(14.5)	14(15.7)		
Senior high school or above	233(43.0)	182(36.1)	10(16.1)	12(13.5)		
Marital status,n(%)					50.657	< 0.001
Married or cohabiting with a spouse	406(74.9)	340(67.5)	28(45.2)	39(43.8)		
Others^a^	136(25.1)	164(32.5)	34(54.8)	50(56.2)		
≥ 3 types of chronic diseases,n(%)	107(19.7)	288(57.1)	30(48.4)	74(83.1)	220.981	< 0.001
Types of medication,n(%)					381.888	< 0.001
0	262(48.3)	19(3.8)	7(11.3)	1(1.1)		
1–3	269(49.6)	412(81.7)	48(77.4)	57(64.0)		
≥ 4	11(2.0)	73(14.5)	7(11.3)	31(34.8)		
ADL score (x ± s)	99.7 ± 10.4	99.4 ± 10.3	74.4 ± 12.6	64.4 ± 13.4	38.830	< 0.001
IADL score (x ± s)	0.8 ± 0.3	1.1 ± 0.3	4.8 ± 1.1	6.1 ± 1.2	76.828	< 0.001
MMSE score (x ± s)	24.2 ± 3.7	23.2 ± 4.2	21.8 ± 4.5	19.0 ± 4.9	30.005	< 0.001
CES-D score(x ± s)	5.2 ± 4.5	6.2 ± 4.7	11.3 ± 5.7	16.7 ± 6.4	86.377	< 0.001
FIvalue (x ± s)	0.07 ± 0.04	0.10 ± 0.04	0.30 ± 0.07	0.33 ± 0.09	91.799	< 0.001
No. of deaths,n(%)	179(33.0)	172(34.1)	35(56.5)	57(64.0)	43.443	< 0.001

*Abbreviations*: *ADL *Activities of daily living, *IADL* Instrumental activities of daily living, *MMSE* Mini-Mental StateExamination, *CES-D* Center for Epidemiologic Studies Depression scale, *FI* Frailty index

^a^Other marital status includes single, separated, divorced, or widowed status

### Influence of hypertension on age-related changes in FI value at baseline

**Fig. 2:**
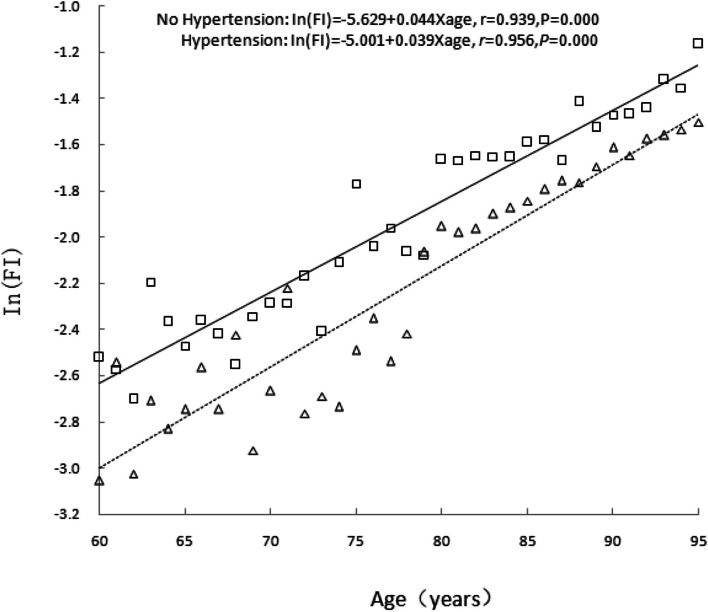
Relationship between age and mean FI value at baseline. The absence of hypertension is indicated by a triangle and dashed line, whereas the presence of hypertension is represented by a square and solid line

The analysis of age-related alterations in the FI value of older adults with hypertension and those without hypertension revealed that the FI value of older adults exponentially increased with age, regardless of the prevalence of hypertension. The following equation was used for calculating the age-related changes in the FI value: ln(FI) = *A + B*×age, where ln(FI) of older adults without hypertension was − 5.629 + 0.044×age (*r* = 0.939, *P* < 0.001) and the ln(FI) of those with hypertension was − 5.001 + 0.039×age (*r* = 0.956, *P* < 0.001). The results showed that the FI value of older adults with hypertension was higher than those without hypertension at any age. However, based on the logarithmic coordinates, the average annual relative increase in the FI value with age in older adults with hypertension was lower than in those without hypertension (*β* = 0.039 *vs. β* = 0.044, *t* = 10.513, *P* < 0.001).This finding indicates that the accumulation rate of health deficits in older adults with hypertension gradually slows down with age when compared with those without hypertension (see Fig. 2 for more details).

### Influence of hypertension on mortality among older adults with different levels of frailty at baseline

**Fig. 3:**
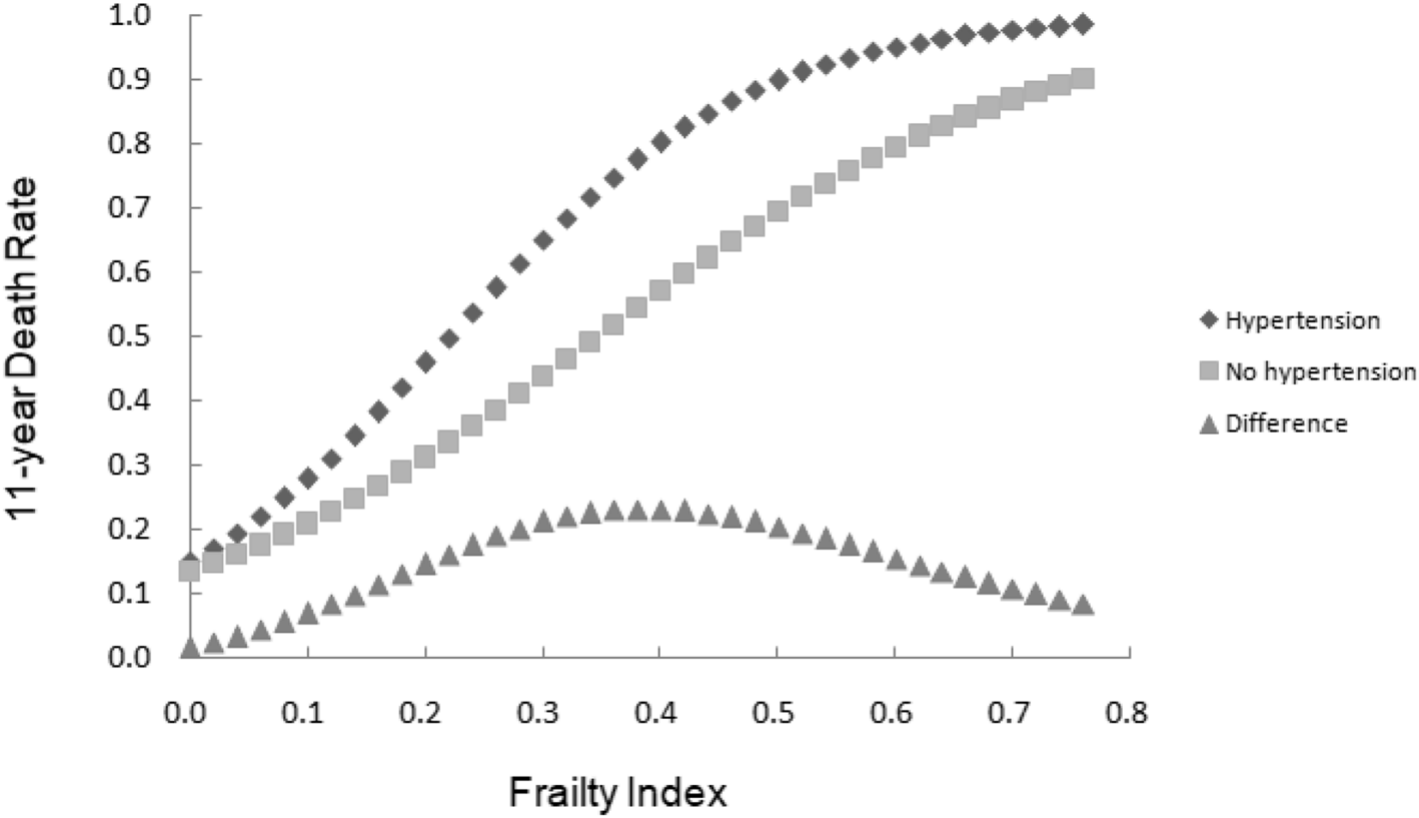
The 11-year death rate as a function of the FI at baseline among older adults with and without hypertension

The assessment of the relationship between baseline FI value and mortality among older adults with hypertension and those without hypertension suggested that the death rate of older adults rises with increasing FI value, regardless of hypertension prevalence. Furthermore, older adults with hypertension were found to have higher mortality rates than those without hypertension at any FI value. The results also showed that the largest difference in death rates between older adults with hypertension and those without hypertension was within the FI values ranging from 0.3 to 0.5, and this difference gradually diminished with increasing frailty degree (see Fig. 3 for further information).

### Kaplan–Meier curves for the proportional survival of older adults with different hypertension and frailty status

**Fig. 4:**
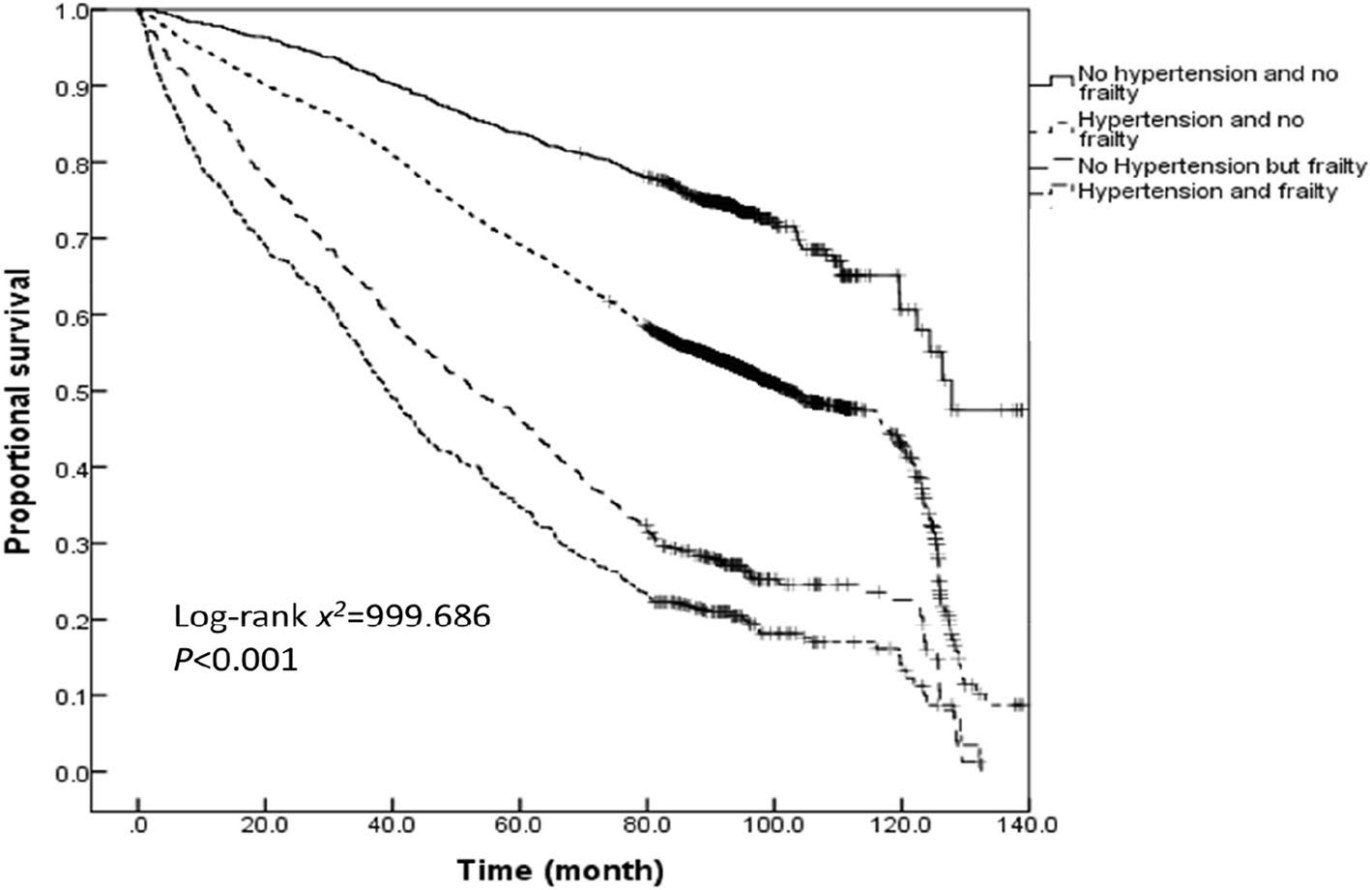
Kaplan–Meier curves for the proportional survival of the total sample population with different hypertension and frailty status

**Fig. 5:**
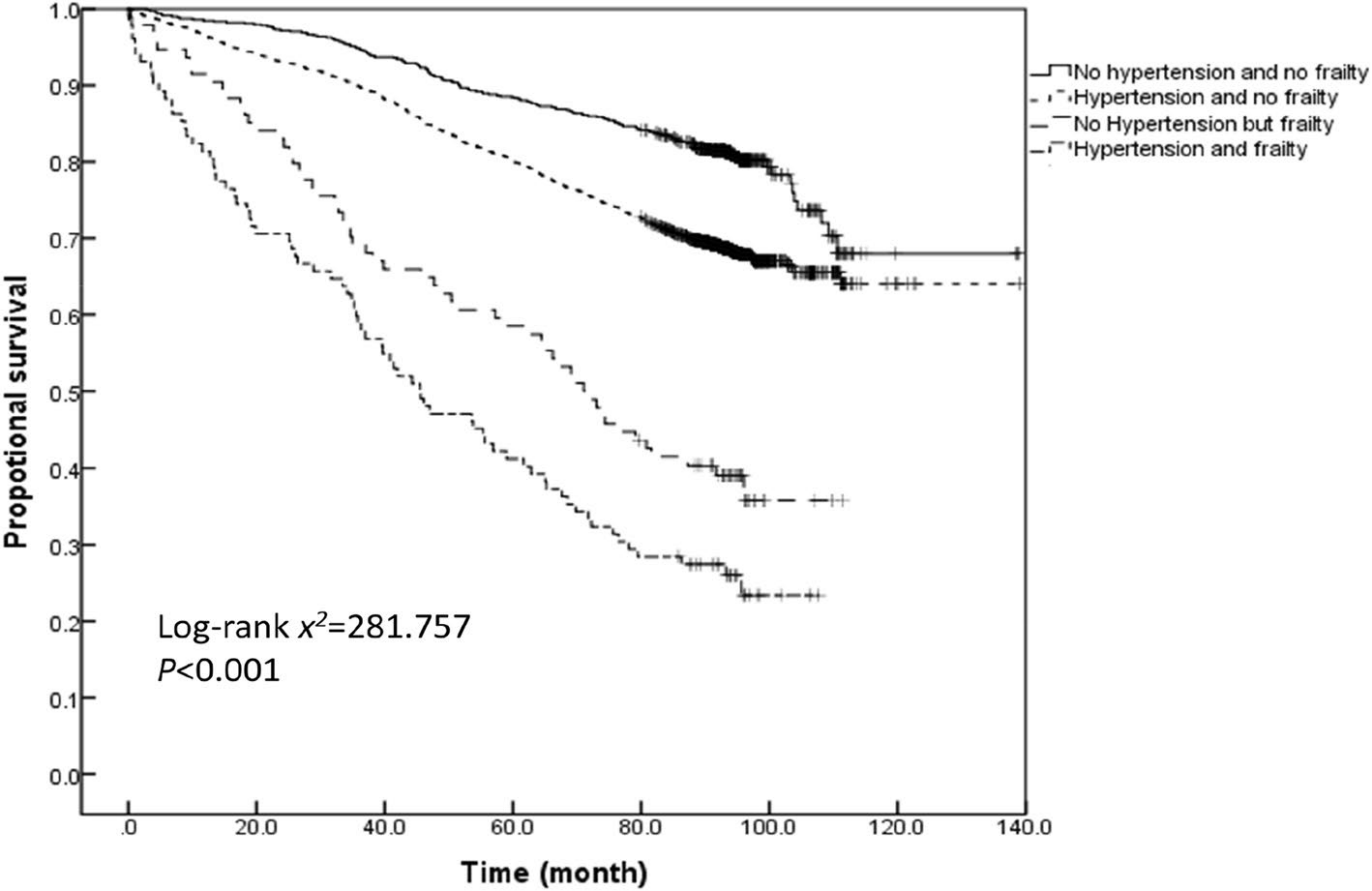
Kaplan–Meier curves for the proportional survival of older adults aged 60–69 years with different hypertension and frailty status

**Fig. 6:**
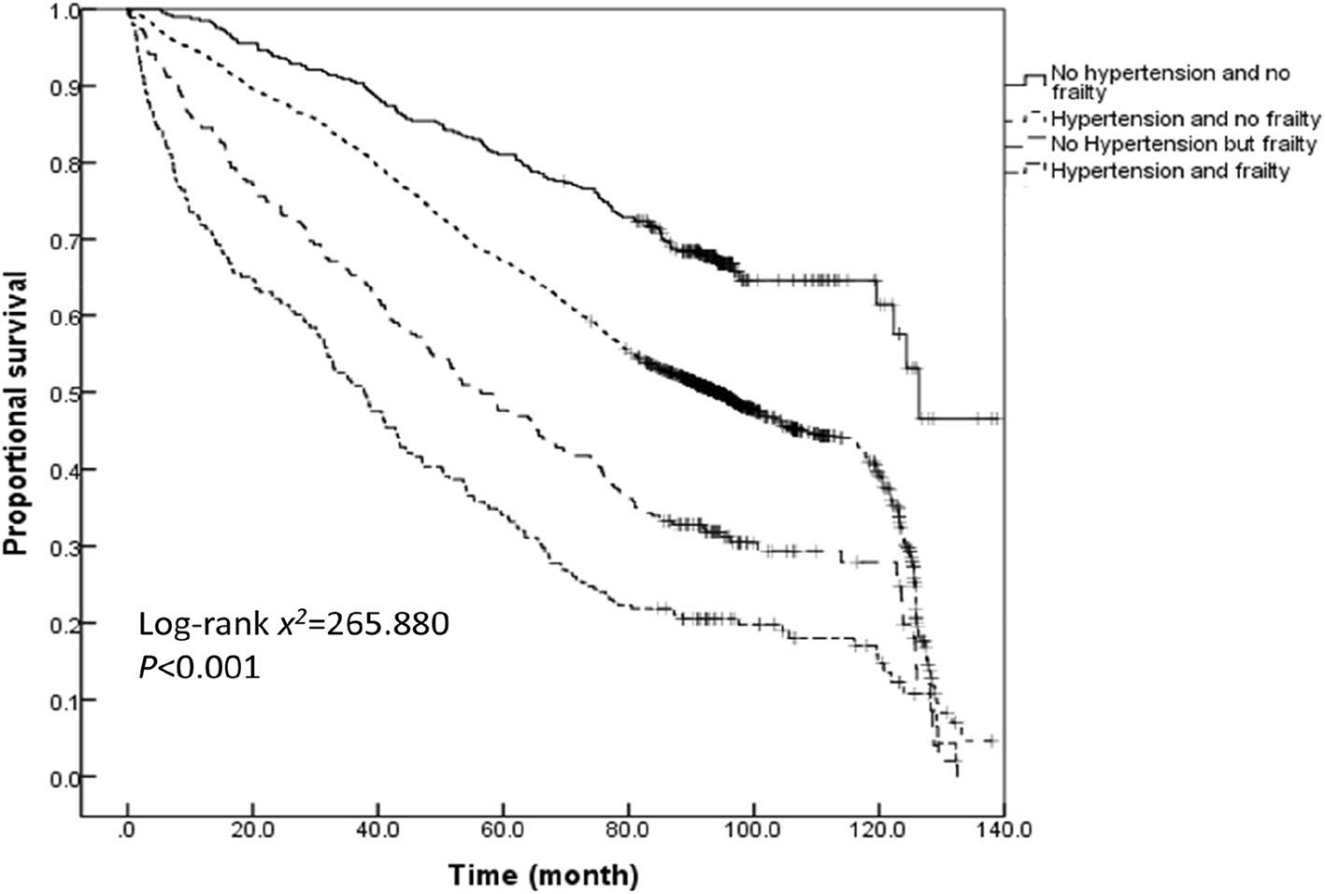
Kaplan–Meier curves for the proportional survival of older adults aged 70–79 years with different hypertension and frailty status

**Fig. 7:**
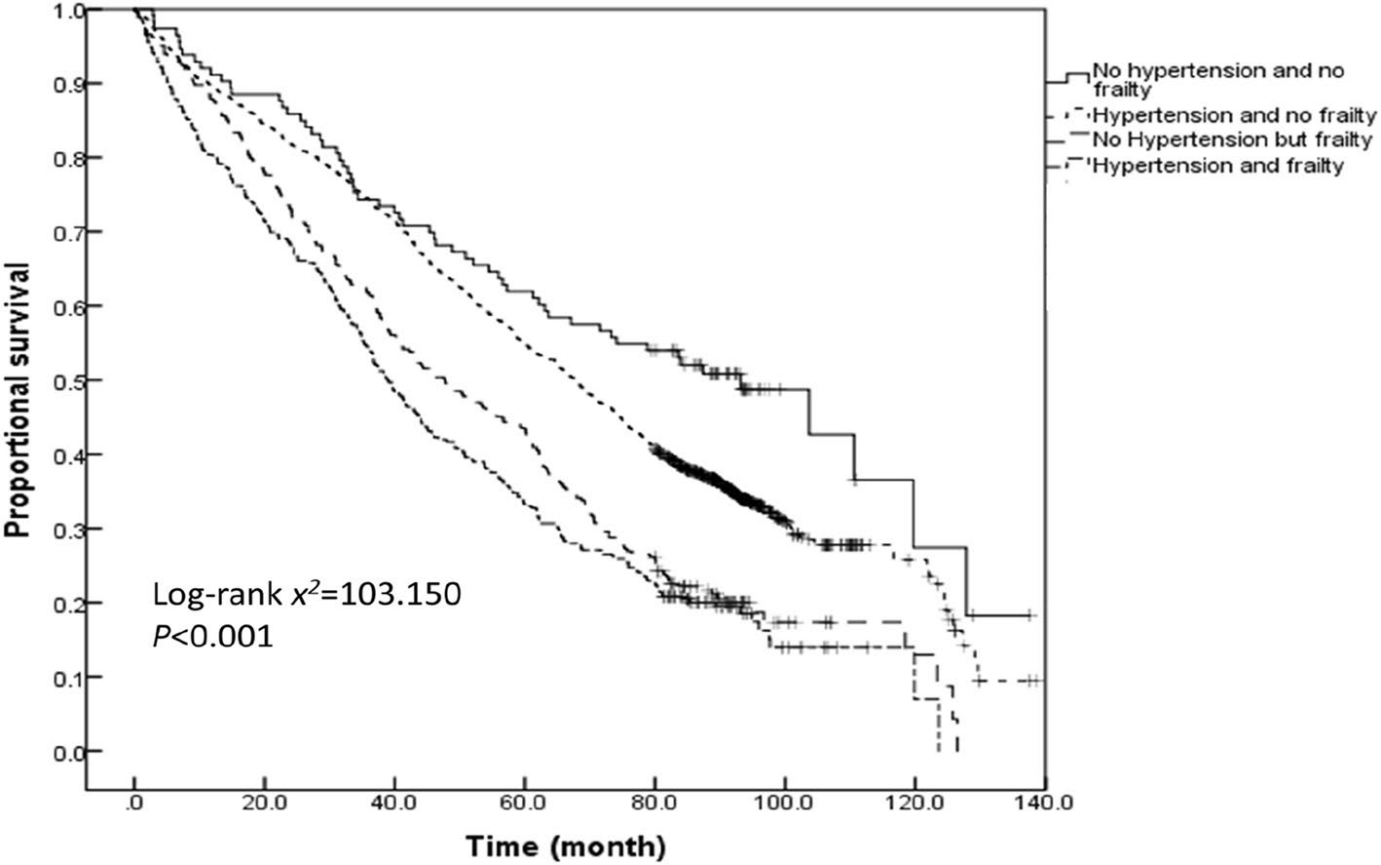
Kaplan–Meier curves for the proportional survival of older adults aged ≥ 80 years with different hypertension and frailty status

The survival time analysis of older adults with varied hypertension and frailty status demonstrated that the average survival time of older adults without hypertension and frailty, those with hypertension and without frailty, those without hypertension but with frailty, and those with both hypertension and frailty during the past 11 years were 110.1 ± 1.6, 88.2 ± 0.6, 61.5 ± 1.8, and 51.0 ± 1.7 months, respectively. According to the Kaplan–Meier curves, the median survival time of older adults having both hypertension and frailty was the shortest (39.0[95%*CI*: 35.6–42.3] months), followed by 52.9 (95%*CI*: 46.6–59.3) months for those having frailty alone, 102.7 (95%*CI*: 98.7–106.8) months for those with only hypertension, and 127.9 (95%*CI*: 113.5–134.7) months for those without hypertension and frailty. A log-rank test further corroborated that the co-occurrence of hypertension with frailty reduces the median survival time of older adults (log-rank *x*^*2*^ = 999.686, *P* < 0.001). The pairwise comparisons also showed significant differences between the survival rates of individuals with varying hypertension and frailty status (all *P* < 0.001). A further comparison of the Kaplan–Meier curves for the proportional survival of the older adults with different hypertension and frailty status stratified by age revealed that the survival rate decreased with the prevalence of hypertension and frailty across all three age groups of 60–69, 70–79 and ≥ 80 years (log-rank *x*^*2*^ = 281.757, 265.880, and 103.150, respectively; all *P* < 0.001). Furthermore, the survival time of the older adults with different hypertension and frailty status was compared pairwise in each of the three age groups.The results demonstrated that the survival rate was significantly different for most pairwise comparisons, except for the survival rate between older adults with both hypertension and frailty and those without hypertension but with frailty who were ≥ 80 years old (*P* = 0.123; see Figs. 4, 5, 6 and 7 for complete details).

### Multivariate Cox regression analysis of the influence of hypertension and frailty status on death risk

We performed multivariate Cox regression analysis using death status and the time of death as dependent variables and adjusting for variables including age, gender (men = 0 or women = 1), education level (primary school = 1, junior high school = 2, senior high school or above = 3), and marital status (married or cohabiting with a spouse = 1 or others = 2),with low values as the control group .The analysis revealed that increasing age led to a higher death risk in older adults, while the death risk in women and older adults who were married or cohabiting with a spouse was lower than that in the respective control groups. Moreover, compared with the older adults without hypertension and frailty, those with hypertension and frailty had the highest mortality risk (*HR* = 1.792, *P* < 0.001), followed by those without hypertension but with frailty (*HR* = 1.484, *P* < 0.001) and those with hypertension but without frailty (*HR* = 1.406, *P* = 0.005). Further analysis according to the age groups indicated that the influence of frailty or hypertension alone on death risk diminished with increasing age, whereas this influence was not significant in older adults ≥ 80 years (all *P* > 0.05).Nevertheless, older adults with hypertension and frailty had an increased risk of death in all age groups (all *P* < 0.001; see Table 2 for more information).

**Table 2:** Multivariate Cox regression analysis of the impact of hypertension and frailty on death risk in older adults according to the different agegroups

Variables	Overall (*n* = 1197)		60–69 years (*n* = 378)		70–79 years (*n* = 415)		≥ 80 years (*n* = 404)	
	*HR*(95%*CI*)	*P*-value	*HR*(95%*CI*)	*P*-value	*HR*(95%*CI*)	*P*-value	*HR*(95%*CI*)	*P*-value
Age	1.048(1.042–1.055)	< 0.001	1.077(1.049–1.106)	< 0.001	1.046(1.025–1.068)	< 0.001	0.994(0.953–1.037)	0.784
Gender	0.721(0.662–0.786)	< 0.001	0.623(0.526–0.739)	< 0.001	0.735(0.646–0.836)	< 0.001	0.850(0.720–1.003)	0.054
Education level^a^								
Junior high school	0.977(0.901–1.046)	0.397	0.923(0.833–1.057)	0.294	0.965(0.849–1.114)	0.326	0.933(0.877–1.366)	0.233
Senior high school or above	0.864(0.812–1.019)	0.183	0.811(0.732–1.016)	0.109	0.852(0.766–1.021)	0.079	0.847(0.807–1.173)	0.128
Marital status	1.371(1.219–1.543)	< 0.001	1.369(1.150–1.630)	< 0.001	1.269(1.059-–1.521)	0.010	1.632(1.107–2.405)	0.013
Hypertension and frailty status^b^								
Hypertension and no frailty	1.406(1.237–1.597)	0.005	1.331(1.159–2.354)	0.021	1.594(1.113–1.744)	0.018	1.174(0.774–1.781)	0.450
No hypertension but frailty	1.484(1.259–1.933)	< 0.001	1.345(1.052–1.721)	0.018	1.511(1.254–1.820)	< 0.001	1.223(0.948–1.578)	0.122
Hypertension and frailty	1.792(1.642–1.957)	< 0.001	2.122(1.777–2.533)	< 0.001	1.821(1.604–2.067)	< 0.001	1.573(1.338–1.850)	< 0.001

*Abbreviations*: *FI* Frailty index

^a^Primary school is the control group

^b^No hypertension and no frailty is the control group

## Discussion

Hypertension and frailty are two critical health issues that often occur concurrently, exhibiting increasing prevalence in the older population [9]. Our study revealed that 49.5% of older adults in a Beijing community experienced hypertension in 2009.Furthermore, women were found to have a higher hypertension prevalence rate than men (54.7% vs. 41.7%), consistent with the findings of the China hypertension survey. The Chinese National Nutrition and Health Survey in 2002 also reported a hypertension prevalence of 49.1% among older adults aged ≥ 60 years [22]. In support of these findings, a previous cross-sectional study conducted from 2012 to 2015 using multistage and stratified random sampling showed a hypertension prevalence of 53.2%.Moreover, the prevalence increased significantly with age and was observed in 51.1% and 55.3% of men and women, respectively [23]. Additionally, we estimated that 12.6% of older adults in the Beijing community exhibited frailty. We further found that frailty showed increased prevalence with age and had a higher prevalence rate in women than in men, in line with the results of previous studies [24, 25]. The comparison of the baseline results in this study revealed that older age, female gender, lower education levels, and widowed status were associated with a higher proportion of hypertension with frailty. Furthermore, a greater number of older adults with hypertension and frailty had three or more chronic diseases, multiple types of medication, a significant decline in the ADL and cognitive function, and an increase in depression scores, all of which were congruent with previous study findings [10, 11, 15]. Therefore, the management of older adults with hypertension and frailty can be enhanced by implementing targeted interventions to delay disease progression, reduce the risk of complications, and minimize adverse outcomes. According to the current study results, older adults with hypertension had a higher prevalence of frailty than those without hypertension (15.0% vs. 10.3%). Moreover, older adults with hypertension exhibited higher baseline FI values than those without hypertension, regardless of their age. A prior meta-analysis indicated that 14.0% (95%*CI*: 0.12–0.17) of patients with hypertension experienced frailty [14]. Similarly, another investigation by Ma et al. [11]showed that the prevalence rate of frailty among older adults with hypertension in China was 19.6%. Additionally, older adults with hypertension and frailty have been found to perform worse in physical, psychological, and social activities than those without frailty. Older adults with hypertension also have a low quality of life, experience several complications, use numerous medications, exhibit physical and cognitive deterioration, and have an elevated risk of falling, all of which contribute to frailty. Therefore, older adults with hypertension are associated with a greater risk of developing frailty [26]. This relationship between hypertension and frailty may be explained by the common pathogenic mechanisms of cardiovascular disease and frailty in older adults with hypertension. For example, the activation of the chronic inflammatory process may serve as the prior pathogenic cause of the coexistence of hypertension with related cardiovascular diseases and frailty. Chronic inflammation is associated with the body’s homeostatic response after exposure to pathogens and involves pathophysiological changes in the nervous, musculoskeletal, endocrine, immune, and hematological systems. Therefore, hypertension and frailty in older individuals can often coexist with multiple diseases and promote each other [27, 28]. Additionally, a previous cross-sectional study found that older adults with pre-frailty and frailty had a higher incidence of atherosclerosis, potentially explaining the relationship between frailty and cardiovascular diseases, such as hypertension [29].

Our study also showed that the death rate of older adults with hypertension was higher than those without hypertension at any degree of frailty. Furthermore, the largest difference in the death rate between these patients was observed within the FI values ranging from 0.3 to 0.5, with this difference gradually decreasing with increasing frailty. Moreover, we performed a Cox regression analysis after adjusting for confounding factors, such as age, gender, education level, and marital status. The results showed that older adults with hypertension and frailty had the highest risk of death. Further analysis according to age revealed that older adults with both hypertension and frailty had an increased death risk in all three age groups, demonstrating that mortality risk is greatly influenced in older adults with hypertension accompanied by frailty than in those with only frailty or hypertension. Our study results suggested that the occurrence and development of hypertension and frailty in older adults are often mutually reinforcing and overlap. Previous research showed that the coexistence and interaction between hypertension and frailty was associated with a significantly increased death rate compared with the presence of hypertension alone [30]. In a large longitudinal aging study in China, Ma et al. [11]. adopted the FI to assess frailty in 1111 patients with hypertension who were ≥ 60 years old and found that compared to patients with hypertension but no frailty, patients with both hypertension and frailty had a higher 8-year death rate. Those study findings, combined with the results of our study, imply that older adults with hypertension and frailty are at higher risk of adverse outcomes than those with hypertension but no frailty. Further, these results suggest that healthcare professionals managing adults with hypertension should pay close attention to the screening and assessment of their frailty status, particularly those with mild or moderate frailty (FI value: 0.3–0.5). In terms of managing hypertensive older adults with frailty, determining the degree of frailty becomes crucial in balancing the benefits and risks of antihypertensive treatment in this specific older population. An earlier meta-analysis found that high systolic and diastolic blood pressure levels could reduce the overall death rate of older patients with frailty, with pulse pressure showing no association with death risk [31]. In support of these observations, Odden et al. [32]. also found that hypertension in older adults with frailty was linked to a lower death rate. However, the European Guidelines have highlighted the risk of overtreatment in older adults with frailty [33], which can potentially increase the incidence of adverse events. Thus, assessing the frailty of hypertensive patients is imperative. The hypertension guidelines published by the International Society of Hypertension in 2020 suggest a target blood pressure of ≤ 140/90 mmHg for individuals who are ≥ 65 years old (if tolerated).Furthermore, the guidelines recommend that the treatment for meeting individualized blood pressure targets should be determined based on the frailty status, independent living capability, and tolerable conditions of the individuals [34]. Moreover, the 2019 Chinese Guidelines for the Management of Hypertension in the Elderly [35] suggest that antihypertensive drug treatment in the oldest-old individuals with frailty should be considered for blood pressure levels ≥ 160/90 mmHg. Furthermore, the target systolic blood pressure should be < 150 mmHg, with further reduction to < 140/90 mmHg if treatment is well tolerated. However, the systolic blood pressure can be as high as 130 mmHg [36]. Additionally, the antihypertensive drugs used in older adults with frailty should be stable, effective, safe, and simple, as well as have only limited adverse effects and good compliance. The drugs should be initially administered in small doses, followed by a gradual increase or the use of small dose combinations.

This study has several limitations that should be considered. First, the data were obtained based on a questionnaire, thereby leading to potential information biases, including memory bias and questionnaire misinterpretation. Second, due to the prospective nature of this study, the older adults lost to follow-up were relatively younger individuals who had left or relocated from the place of the survey. This possible bias due to the loss to follow-up may have affected the study results. Third, the cause of death in the older adults was not collected in this study due to the COVID-19 pandemic restrictions, we could not conduct home visits during the follow-up period in 2020; therefore, we relied on telephone interviews with the family members, local neighborhood committees, or local public security agencies to obtain information on death (yes or no) and the time of death. Thus, the influence of factors other than hypertension and frailty on death cannot be excluded. Finally, the data on blood pressure control were not acquired during the follow-up survey, potentially affecting the findings. Therefore, future studies with further improvements in the questionnaire and inclusion of relevant information to enable a more comprehensive analysis are warranted.

## Conclusions

Frailty is prevalent among older adults. Moreover, frailty and hypertension have been shown to interact with each other, with frailty exacerbating the outcomes of older individuals with hypertension. Therefore, strengthening our understanding of frailty and promoting its assessment in older adults, particularly those with hypertension, is crucial. Additionally, hospitals should prioritize and strengthen the monitoring as well as facilitate the early-targeted intervention of frailty in older patients with both hypertension and frailty. In the context of community settings, community health service centers should intensify the screening and follow-up of frailty status in older adults with hypertension, as well as offer timely guidance to the patients and their families. All these measures will contribute toward preventing the occurrence of frailty-related adverse outcomes and mitigating the medical burden of hypertension accompanied by frailty in the older population.

### Supplementary Information

**Supplementary material 1.**

## Data Availability

The data are available from the corresponding author on reasonable request.
