# Supplementary material for: Interaction between hypertension and frailty and their impact on death risk in older adults: a follow-up study
Source: BMC Geriatr. 2024 Feb 24;24:187. doi: 10.1186/s12877-024-04793-w (PMC10893602; doi:10.1186/s12877-024-04793-w)
Supplement: Supplementary file 1 — Supplementary material 1. [file 12877_2024_4793_MOESM1_ESM.docx]

**Additional Table 1** **Variables for the construction of the frailty index and their scoring system**

| **Variables** | **Scoring system** |
| --- | --- |
| **Comprehensive geriatric assessment** |  |
| Falls | No=0, Yes=1 |
| Urinary incontinence | Never=0, ≤1 time/week=0.25, 2–3 times/week=0.5, Approximately 1 time/day=0.75, Several times/day=1 |
| Pain | None=0, Tolerable without affecting any activities=0.33, Tolerable but affecting some activities=0.66, Intolerable but still able to make phone calls/watch TV/perform other activities=1 |
| Constipation | No=0, Yes=1 |
| Weight loss | No=0, Yes=1 |
| Sleep disorder | No=0, Yes=1 |
| Use of sleep aids | No=0, Yes=1 |
| **Visual and hearing assessment** |  |
| Vision | >4 m=0, 1–3 m=0.5, ≤1 m=1 |
| Hearing | Completely clear=0, Not very clear= 0.5, Not clear at all = 1 |
| **Walking balance function** |  |
| Use of walking aids | No=0, Yes=1 |
| Able to walk 400 m independently | Yes=0, No=1 |
| Static balance test | >10s=0, ≤10s=0.5, Unable to complete=1 |
| Dynamic balance test | >10s=0, ≤10s=0.5, Unable to complete=1 |
| 5 times sit-to-stand test | <10s=0, ≥10s=0.5, Unable to complete=1 |
| Up-and-go test | <12s=0, ≥12s=0.5, Unable to complete=1 |
| **Diseases and medication** |  |
| Chronic diseases(14 types)^a^ | Each no=0, Each yes=1 |
| Number of medications | Infrequent medications=0, 1–3 medications=0.5, ≥4 medications=1 |
| **Assessment of activities of daily living** |  |
| ADL | 100 points=0, 75–95 points=0.25, 50–70 points=0.5, 25–45 points=0.75, 0–20 points=1 |
| IADL | ≤5 points=0, >5 points=1 |
| **Cognition and emotion** |  |
| Memory loss | No=0, Yes=1 |
| Emotional instability | Never=0, Sometimes=0.5, Often=1 |
| MMSE^b^ | Above cut-off value indicating normal cognition=0, Below cut-off value signifying a cognitive deficit=1 |
| **Depression assessment** |  |
| CES-D | <10 points=0, ≥10 points=1 |

Note: ADL: activities of daily living, IADL: instrumental activities of daily living, MMSE: Mini-Mental State Examination, CES-D: Center for Epidemiologic Studies Depression Scale (simplified version).

^a^The14 types of chronic diseases include diabetes, heart disease, anemia, hyperlipidemia, sleep apnea syndrome, gastrointestinal disease, cerebrovascular disease, dementia, tumor, intervertebral disc protrusion, thyroid disease, osteoporosis, osteoarthritis, and arthrolithiasis (except for hypertension).

^b^MMSE cut-off value is related to the level of education, with 17 points for no education, 20 for <6 years of education, and 24 for >6 years of education.
